# Meetings of Societies

**Published:** 1951-07

**Authors:** 


### Meetings of Societies

### Bristol Medico-Chirurgical Society

The Seventh Meeting of the session was held in the Physics Lecture Theatre, Royal Fort, on Wednesday, 11th April, 1951. Dr. Howard K. Gray, M.D., F.A.C.S., gave an address on **"The Mayo Clinic"**, illustrated by a film. We are not allowed to publish what he said.

The Eighth Meeting of the session was held on Wednesday, 9th May, 1951. Dr. J. Apley and Professor R. Milnes Walker described **"The Diagnosis and Treatment of Congenital Heart Disease"**. We hope to publish their papers in our next issue.

Complaints have been heard that members of the Society do not receive copies of the Balance Sheet before the Annual General Meeting at which they are asked to approve it. So that by the time it appears in print, as part of the proceedings, the following January, it is too late to discuss details: for example, why the number of subscriptions is less than the number of members, It is therefore hoped to publish the Balance Sheet in future with the October issue before the date of the meeting.

### The South-Western Laryngological Association

The Spring Meeting of the Association was held in the South Devon and East Cornwall Hospital, Greenbank Road, Plymouth, on Saturday, 7th April, 1951. It was preceded by a lunch at the Grand Hotel: but this hardly seems an adequate explanation of the difficulty experienced in finding the meeting place. The local members exhibited twenty cases: six cases of malignant disease of upper jaw, treated by operation and irradiation; three cases of frontal osteomyelitis secondary to sinus disease; six cases of laryngeal or pharyngeal cancer recently treated; and two or three other conditions. A lively discussion took place after the clinical meeting. Mr. A. Leigh, F.R.C.S., was elected President for the ensuing session. The next meeting will be in Birmingham on 14th October.

### British Medical Association

### Bath, Bristol and Somerset Branch

A meeting of this Branch was held at Taunton on 10th May, Dr. Petty, President of the Branch, in the Chair. Mr. Belsey spoke on "**Recent Advances in Thoracic Surgery**". He described the effect on infection of the lungs of chemotherapy and physiotherapy. Taking empyema as an example, he pointed out that it now often presents as a sterile condition requiring surgery—thoracotomy and excision of the pleura. He said that because of improved dental hygiene and greater care by dentists, lung abscess was becoming rare: segmental removal rather than lobectomy is the modern surgical treatment, and bronchiectasis and compensatory emphysema were avoided by phrenic section. In pulmonary tuberculosis segmental excision had replaced lobectomy, though thoracoplasty was still necessary in some cases. Cancer of the lung has increased, and it is more often recognized by the general practitioner from the clinical signs than by mass radiography. The early symptoms are a non-productive cough and pain in the chest: the X-ray signs of atelectasis and hilal abnormality are important. Treatment is surgical for X-rays are ineffective. Mr. Belsey also stressed the great value of bronchoscopy; changes in the motility of the bronchus are of great significance. Finally, he discussed strictures of the oesophagus, carcinoma and hiatal hernia, demonstrated by excellent X-ray films; answered many questions and encouraged discussion.

A clinical meeting of the Branch was held at Southmead Hospital, Bristol, on Wednesday, 6th June, the President in the Chair: 152 members attended. Over forty very interesting cases were shown, covering a wide range of conditions, by the following members of the hospital staff: Professor Neale, Drs. Beryl Corner, Airth, J. Apley,

Curwen, J. M. Naish, J. MacRae, A. L. Roberts, K. E. Pearson, P. Phillips, R. Warin, Orr Ewing and W. Lewis; Messrs. Brown, G. Scarff, J. A. Pocock, MacPherson, A. Miller and Capper.

Despite a very warm summer evening, there was an excellent attendance and after the meeting very pleasant refreshments were served. Great credit must go to the nursing and catering staff of the hospital, to whom we were indebted for the use of the buildings and particularly to the Out-Patients Sister, Sister Deal, for her organization and handling of the cases.

### Bristol Division

The Annual General Meeting of this Division was held in the large theatre of the Wills Physics Department of Bristol University (Royal Fort) on Wednesday, 2nd May. The great importance of this meeting had been impressed on all members and not very many less than 10 per cent. attended. The following officers were elected for 1951–52:

*Chairman.* Dr. T. Milling; *Chairman-elect*, Mr. R. G. Paul; *Secretaries*, Drs. W. H. Hayes and R. C. Wofinden; *Treasurer*, Dr. C. Dix; *Representatives*, Drs. P. Phillips, A. G. Heron, T. Milling, W. G. Nott, W. Woolley.

The meeting adopted a special vote of thanks to Dr. H. M. Golding for his very valuable services as Secretary during the last ten years.

